# Intrauterine Growth Restriction Increases TNF**α** and Activates the Unfolded Protein Response in Male Rat Pups

**DOI:** 10.1155/2014/829862

**Published:** 2014-04-06

**Authors:** Emily S. Riddle, Michael S. Campbell, Brook Y. Lang, Ryann Bierer, Yan Wang, Heidi N. Bagley, Lisa A. Joss-Moore

**Affiliations:** ^1^Division of Nutrition, University of Utah, Salt Lake City, UT 84158, USA; ^2^Department of Pediatrics, University of Utah, Salt Lake City, UT 84158, USA; ^3^Division of Neonatology, University of Utah, P.O. Box 581289, Salt Lake City, UT 84158, USA

## Abstract

Intrauterine growth restriction (IUGR) programs adult disease, including obesity and insulin resistance. Our group previously demonstrated that IUGR dysregulates adipose deposition in male, but not female, weanling rats. Dysregulated adipose deposition is often accompanied by the release of proinflammatory signaling molecules, such as tumor necrosis factor alpha (TNF**α**). TNF**α** contributes to adipocyte inflammation and impaired insulin signaling. TNF**α** has also been implicated in the activation of the unfolded protein response (UPR), which impairs insulin signaling. We hypothesized that, in male rat pups, IUGR would increase TNF**α**, TNFR1, and components of the UPR (Hspa5, ATF6, p-eIF2**α**, and Ddit3) prior to the onset of obesity. We further hypothesized that impaired glucose tolerance would occur after the onset of adipose dysfunction in male IUGR rats. To test this hypothesis, we used a well-characterized rat model of uteroplacental insufficiency-induced IUGR. Our primary findings are that, in male rats, IUGR (1) increased circulating and adipose TNF**α**, (2) increased mRNA levels of UPR components as well as p-eIF2a, and (3) impaired glucose tolerance after observed TNF**α** increased and after UPR activation. We speculate that programmed dysregulation of TNF**α** and UPR contributed to the development of glucose intolerance in male IUGR rats.

## 1. Introduction

Uteroplacental insufficiency secondary to maternal hypertension is a common complication of pregnancy and is a leading cause of intrauterine growth restriction (IUGR) in developed countries [1–3]. In uteroplacental insufficiency-induced IUGR, reductions in blood flow to the fetus restrict growth and inhibit the fetus from achieving its* in utero* genetic growth potential.

IUGR programs individuals to develop adult disease, including obesity and insulin resistance [4–8]. Although IUGR infants are born smaller than their appropriately grown counterparts, the rate of adipose deposition in IUGR infants is accelerated throughout childhood and favors deposition of visceral adipose tissue (VAT) over subcutaneous adipose tissue (SAT) [9]. Not surprisingly, comorbidities of obesity such as insulin resistance and type 2 diabetes are also prevalent in former IUGR individuals [4, 6].

Using a well-defined rat model of IUGR, our group demonstrated that IUGR increases the accumulation of VAT relative to SAT in male, but not female, weanling rat pups [10]. Sex-specific dysregulated adipose deposition in our model is accompanied by molecular changes in adipose tissue as well as elevated muscle triglycerides [11]. Additionally, the observed adipose dysfunction takes place* prior* to the onset of overt obesity, when IUGR rat pups still weigh less than control rat pups.

Adipocyte dysfunction is generally characterized by the overexpansion of adipose tissue and concomitant release of proinflammatory signaling molecules [12]. As a result, adipocytes lose their ability to efficiently sequester and store lipid leading to elevated circulating lipids, as well as ectopic lipid deposition in liver and muscle. The combination of adipose tissue inflammation and ectopic lipid deposition contributes to widespread insulin resistance [12].

Tumor necrosis factor alpha (TNF*α*) contributes to adipocyte inflammation and insulin resistance [13]. TNF*α* is a proinflammatory cytokine produced within adipose tissue. TNF*α* activates a proinflammatory signaling cascade and inhibits insulin receptor signaling [14, 15]. TNF*α* is synthesized as a monomeric transmembrane protein (mTNF*α*) that is active in its homotrimeric form. This homotrimer can be cleaved to produce a soluble signaling molecule (sTNF*α*) [16]. Both sTNF*α* and mTNF*α* can bind and activate tumor necrosis factor receptor 1 (TNFR1). Evidence suggests that TNFR1 is the key mediator of TNF*α*'s activity in the majority of cell types [17]. Various downstream targets, including the transcription factor Jun N-terminal kinase (JNK), can be activated in response to TNFR1 activation [17, 18].

Recently, TNF*α* has been implicated in the activation of the unfolded protein response (UPR) [19]. The UPR is a cell survival mechanism activated in response to cellular stress and accumulation of improperly folded protein products in the endoplasmic reticulum (ER) [20]. Members of the heat shock family of proteins, including heat shock protein 5 (Hspa5), recognize unfolded proteins in the ER. Recognition of unfolded proteins triggers the activation of ER transmembrane proteins, including protein kinase RNA-like endoplasmic reticulum kinase (PERK) and activating transcription factor 6 (AFT6). Activation of PERK reduces the number of new proteins produced via the phosphorylation and inactivation of eukaryotic translation initiator factor 2*α* (eIF2*α*). Activation of ATF6 increases the transcription of ER chaperone proteins, including Hspa5, as well as genes involved in ER-associated degradation. In the event that these compensatory mechanisms are insufficient to restore homeostasis, cell apoptosis is mediated through varying mechanisms including the eIF2*α* activation of the growth-arrest transcription factor DNA-damage inducible transcript 3 (Ddit3). A downstream effect of ER stress and activated UPR is reduced insulin receptor signaling [13, 21].

Despite the presence of adipocyte dysfunction in IUGR rats, the effect of IUGR on the TNF*α* system and UPR in relation to the development of impaired glucose homeostasis is unknown. We hypothesized that, in male rat pups, IUGR would increase TNF*α*, TNFR1, and components of the UPR (Hspa5, ATF6, p-eIF2*α*, and Ddit3) prior to the onset of obesity. We further hypothesized that impaired glucose homeostasis would occur after the onset of adipose dysfunction in male IUGR rats. To test this hypothesis, we used a well-characterized rat model of uteroplacental insufficiency-induced IUGR [10, 22, 23].

## 2. Materials and Methods

### 2.1. Animals

All procedures were approved by the University of Utah Animal Care Committee and are in accordance with the American Physiological Society's guiding principles [24]. The rat uteroplacental insufficiency model of IUGR has been previously described in detail [10, 22, 23]. Briefly, on day 19 of gestation, pregnant Sprague-Dawley rats were anesthetized with intraperitoneal xylazine (8 mg/kg) and ketamine (40 mg/kg). Both uterine arteries were ligated, giving rise to IUGR pups. Control dams underwent identical anesthetic procedures. Rat pups rendered IUGR in this model are ~25% lighter than control rat pups at birth [10].

After maternal rats delivered spontaneously at term, pups were weighed and litters were randomly culled to six pups. Pups remained with the dam and were fed via lactation until postnatal day 21 (d21). On d21, rat pups were killed and subcutaneous and retroperitoneal (a representative visceral depot) adipose tissue was dissected, and serum was collected. All samples were flash frozen in liquid nitrogen and stored at −80°C.

A separate set of rats was used for glucose tolerance experiments. For these experiments, one male and one female rat from each control and IUGR litter were randomly selected for d21 glucose tolerance and one male and one female from each control and IUGR litter were randomly selected for d45 glucose tolerance studies. For RT-PCR and western blot experiments, 6 nonsibling rat pups were used per group. For GTT and HOMA-IR, 4 nonsibling rat pups were used per group.

### 2.2. Serum TNF*α*

Serum TNF*α* was quantified using an enzyme linked immunosorbent assay (ELISA) (BD OptEIA rat TNF*α* ELISA kit (BD, San Diego, CA)) according to the manufacturer's instructions.

### 2.3. Real-Time RT-PCR

Real-time reverse transcriptase polymerase chain reaction (RT-PCR) was used to evaluate mRNA abundance of adipose TNF*α* and TNFR1 as well as Hspa5, Atf6, and Ddit3 as previously described [10, 22, 23]. The following assay-on-demand primer/probe sets were used: TNF*α* Rn99999017 m1, TNFR1 Rn00565310 m1, Atf6 Rn01490854_m1, Ddit3 Rn00492098_g1, and Hspa5 Rn00565250_m1 (Applied Biosystems, Foster, CA). GAPDH was used as an internal control (GAPDH primer and probe sequences; forward: CAAGATGGTGAAGGTCGGTGT, reverse: CAAGAGAAGGCAGCCCTGGT, and probe: GCGTCCGATACGGCCAAATCCG).

### 2.4. Immunoblot

Adipose tissue levels of TNF*α*, TNFR1, eIF2*α*, and phospo-eIF2*α* protein were quantified using immunoblot as previously described [10, 22, 23]. The following primary antibodies were used: Phospho-eIF2*α* (3597S, Cell Signaling Technology), eIF2*α* (9722S, Cell Signaling Technology), TNF*α* (3707, Cell Signaling Technology), and TNFR1 (T9161-06G, US Biological). GAPDH was used as an internal control (2118L, Cell Signaling Technology).

### 2.5. Glucose Tolerance Test and HOMA-IR

For intraperitoneal glucose tolerance tests (IP-GTT), pups on d21 were fasted for 6 hours prior to procedure and d45 rats were fasted for 12 hours prior to procedure. Rats had access to ad libitum water throughout fasting and procedure. After a fasting glucose level was acquired, dextrose solution (2 mg/kg) (Sigma Chemical Co, St Louis, MO) was administered via I.P. injection. At fasting, 15, 30, 60, and 90 minutes after IP-glucose load, blood was obtained via tail venipuncture. Glucose levels (mg/dL) were obtained in real time with a glucometer (Accu-Chek Aviva, Indianapolis, IN).

The homeostasis model of assessment-insulin resistance (Homa-IR) was used as an indicator of insulin resistance in late adolescent male and female rats. Homa-IR was calculated using the US formula: (fasting glucose (mg/dL) ∗ fasting insulin (uU/mL))/405 [25]. Fasting insulin (ng/mL) was quantified using an ELISA kit (Crystal Chem Inc., Downers Grove, IL).

### 2.6. Statistical Analysis

Serum and mRNA data are presented as means ± SEM. Protein data are presented as IUGR relative to sex-matched controls ± SEM. Statistical significance was determined using ANOVA using the StatView 5 software package (SAS Institute, Inc.). *P* ≤ 0.05 was considered significant.

## 3. Results

### 3.1. Pup Weights

On d21, male and female IUGR rat pups weigh significantly less than sex-matched control rat pups. On d45, male rat pups weigh less than male control, while female IUGR rat pups weigh the same as female control rat pups (Table 1).

### 3.2. Serum TNF*α*

**Figure 1:**
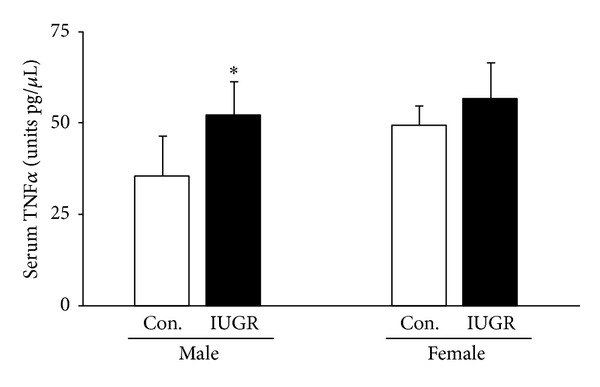
Serum TNF*α*. IUGR increased serum TNF*α* in male rats. Results are control (white bars) and IUGR (black bars). Errors are SD. *n* = 6, *P ≤ 0.05.

Circulating TNF*α* levels were measured in male and female rat pups on d21. In male rat pups, IUGR significantly increased serum TNF*α* relative to male controls (*P* = 0.02). No differences were detected in female rats (Figure 1).

### 3.3. TNF*α* mRNA and Protein

**Figure 2:**
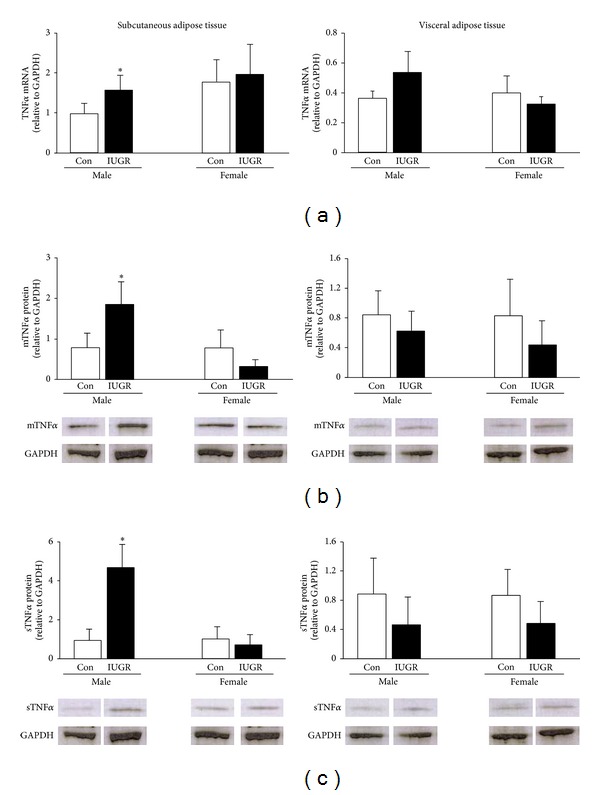
TNF*α* mRNA and protein levels. IUGR increases subcutaneous TNF*α* mRNA and protein levels in male rats. Results are control (white bars) and IUGR (black bars). Errors are SD. *n* = 6, *P ≤ 0.05.

Levels of TNF*α* mRNA as well as sTNF*α* and mTNF*α* protein abundance were measured in SAT and VAT of male and female rat pups on d21. In male rat pups, IUGR significantly increased TNF*α* mRNA in SAT relative to male controls (*P* = 0.04). IUGR did not significantly alter TNF*α* mRNA in SAT of female rat pups or TNF*α* mRNA in VAT of either sex (Figure 2(a)). In male rat pups, IUGR significantly increased mTNF*α* protein abundance (*P* = 0.004) and sTNF*α* protein abundance (*P* < 0.001) in SAT relative to male controls. IUGR did not significantly alter mTNF*α* protein abundance or sTNF*α* protein abundance in SAT of female rats or in VAT of either gender (Figures 2(b) and 2(c)).

### 3.4. TNFR1 mRNA and Protein

**Figure 3:**
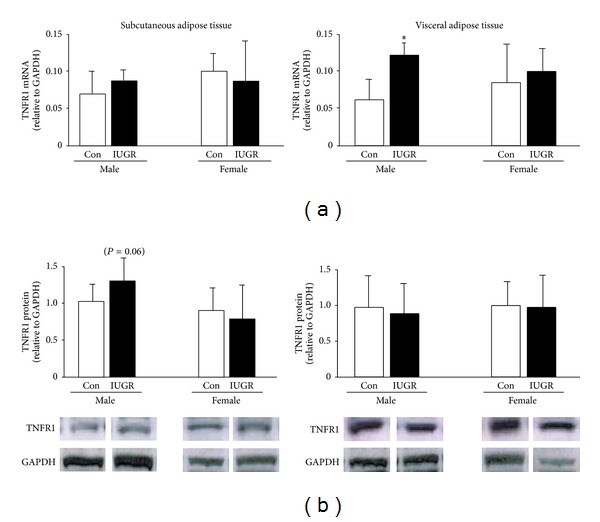
TNFR1 mRNA and protein levels. IUGR increases visceral TNFR1 mRNA levels in male rats. Results are control (white bars) and IUGR (black bars). Errors are SD. *n* = 6, *P ≤ 0.05.

Levels of TNFR1 mRNA and protein abundance were measured in SAT and VAT of male and female rat pups on d21. In male rat pups, IUGR significantly increased TNFR1 mRNA in VAT relative to male controls (*P* = 0.02) (Figure 3(a)). IUGR did not significantly alter TNFR1 mRNA in VAT of female rats or in SAT of either gender. TNFR1 protein abundance was not significantly altered in male or female VAT or SAT (Figure 3(b)).

### 3.5. Hspa5, Atf6, and Ddit3 mRNA and eIF2*α* Protein Phosphorylation

**Figure 4:**
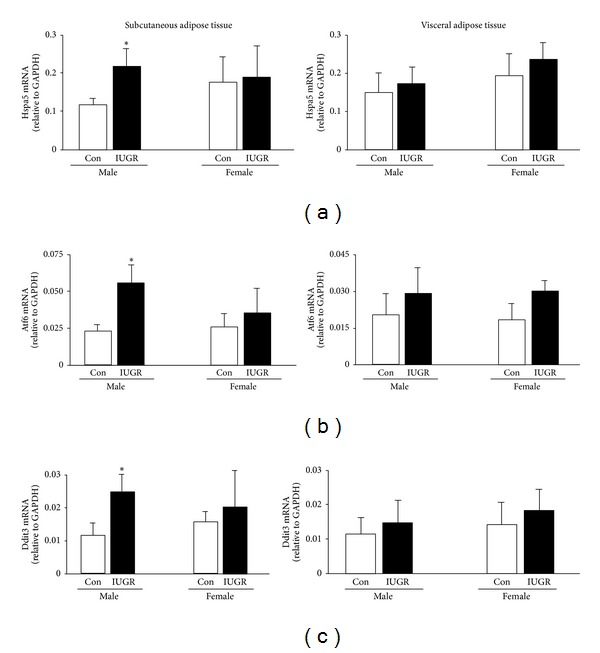
UPR mRNA levels. IUGR increases subcutaneous Hspa5, Atf6, and Ddit3 mRNA levels in male rats. Errors are SD. *n* = 6, *P ≤ 0.05.

**Figure 5:**
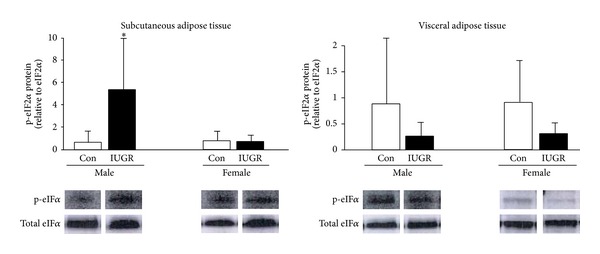
Phospho-eIF2*α* levels. IUGR increases subcutaneous phospho-eIF2*α* levels in male rats. Errors are SD. *n* = 6, *P ≤ 0.05.

In order to assess activation of the unfolded protein response, levels of Hspa5, Atf6, and Ddit3 mRNA and eIF2*α* protein phosphorylation were measured in SAT and VAT of male and female rat pups on d21. In male rat pups, IUGR significantly increased mRNA levels of Hspa5 (*P* = 0.001), Atf6 (*P* < 0.001), and Ddit3 (*P* < 0.001) in SAT relative to male controls. IUGR did not significantly affect Hspa5, Atf6, and Ddit3 mRNA levels in SAT of female rats or in VAT of either sex (Figures 4(a), 4(b), and 4(c)). In male rat pups, IUGR significantly increased the ratio of phosphorylated eIF2*α* protein to unphosphorylated eIF2*α* protein in SAT relative to male control (*P* = 0.04). IUGR did not significantly alter the ratio of phosphorylated eIF2*α* protein to unphosphorylated eIF2*α* protein in male VAT or in any female depot when compared to sex-matched controls (Figure 5).

### 3.6. IP-GTT and HOMA-IR

**Figure 6:**
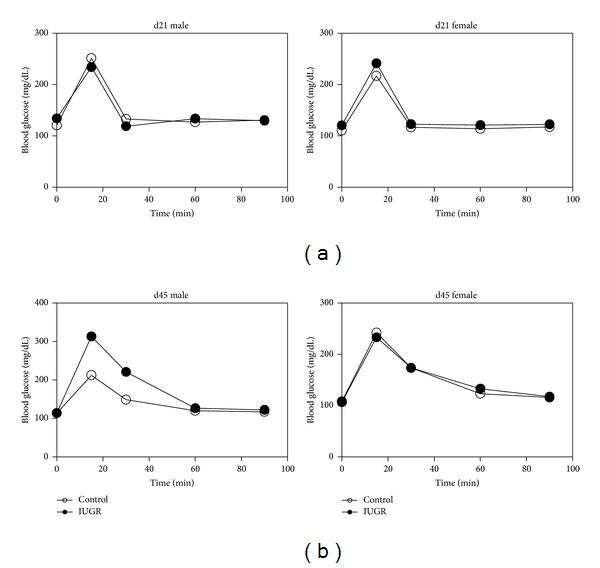
Glucose tolerance tests. IUGR impairs glucose tolerance in d45 in male rats. Results are control (white circles) and IUGR (black circles) Errors are SD. *n* = 4, *P ≤ 0.05.

**Figure 7:**
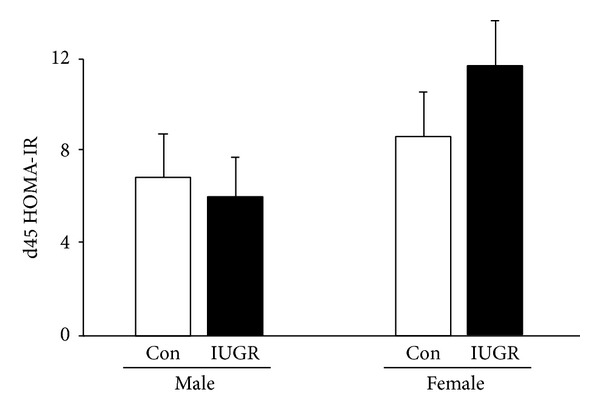
HOMA-IR. IUGR does not alter HOMA-IR in male or female rats at d45. Results are control (white bars) and IUGR (black bars). Errors are SD. *n* = 4.

Since both TNF*α* and the UPR are implicated in glucose tolerance, we performed an IP-GTT and HOMA-IR measurement in male and female control and IUGR rats on d21 and again on d45. IUGR did not impair glucose tolerance in male or female rat pups on d21 (Figure 6(a)). However, on d45, in male rats, IUGR impaired glucose tolerance at 15 (*P* = 0.04) and 30 (*P* = 0.02) minutes after IP glucose dose relative to male control rats (Figure 6(b)). IUGR did not affect glucose tolerance in d45 female rats. IUGR did not alter Homa-IR in male or female rats on d21 or d45 (Figure 7).

## 4. Discussion

The novel results of our study demonstrate that IUGR dysregulates the TNF*α* system and activates the UPR in an adipose depot and sex-specific manner prior to the onset of obesity and impaired glucose tolerance. The majority of molecular effects were confined to the SAT of male rat pups, with female rat pups being relatively unaffected. Importantly, in weanling (d21) rat pups, the dysregulated TNF*α* system and UPR activation occurred in the context of normal glucose tolerance. However, by d45, impaired glucose tolerance was detectable in male, but not female, rats. Collectively, our results suggest that, in male rats, IUGR programs increased adipose inflammation, cellular stress, and UPR activation with subsequent impaired glucose tolerance.

Results from this study expand upon the characterization of adipocyte dysfunction in IUGR rats. Our observation of significantly elevated serum TNF*α* in male rat pups at d21 indicates the presence of systemic inflammation. On d21, IUGR rat pups from our model of IUGR still weigh approximately 25% less than controls, and overt obesity is not yet evident. Increased inflammation in IUGR male SAT before the onset of obesity suggests early programmed adipose dysfunction.

Interestingly, the TNF*α* system in VAT of male rats was unaffected by IUGR. A potential explanation for this may be that adipocytes in the visceral depot are smaller in size than those in the subcutaneous depot [26]. Hypertrophy has been shown to be the preferential mode of expansion in SAT, while hyperplasia is favored in VAT [26]. In humans, adipocyte size is positively correlated with TNF*α* levels [27, 28].

Our study also demonstrated that increased SAT TNF*α* production in male IUGR rat pups is associated with activation of the UPR. TNF*α* has previously been shown to induce UPR through PERK-mediated eIF2*α* phosphorylation and ATF6 [19]. Results from our study demonstrate that adaptive responses, including ATF6 production and eIF2*α* phosphorylation, are increased in male IUGR rat pups. The apoptosis phase mediator, Ddit3, is also significantly increased in male IUGR rat pups. Recent studies suggest that adipocyte apoptosis may play a key role in adipose tissue metabolic dysregulation and macrophage infiltration [29]. In this study, we did not assess apoptosis of adipose cells. However, quantification of adipocyte apoptosis will be an important future step to understand the consequences of activated UPR in IUGR adipose tissue.

We showed that IUGR impairs glucose tolerance in male rats on d45, with no alterations in glucose tolerance being observed on d21. Our data are consistent with previous studies using a similar model of uteroplacental insufficiency-induced IUGR in which IUGR-induced fasting hyperglycemia and hyperinsulinemia were evident by day 70, with an overt diabetic phenotype by day 100 [30]. Elevated glucose has been observed as early as day 7 in IUGR rats; however, postnatal nutrition may have differed from our study, as litters were reared with different number of pups [30]. Our study is the first study to show early signs of glucose intolerance in IUGR rats following elevated TNF*α* and activation of the UPR. Both TNF*α* and the UPR may induce cellular insulin resistance through activation of the JNK signaling cascade [31]. JNK decreases the action of insulin receptor substrate-1, an important intermediate in insulin signaling, through serine phosphorylation [32]. Thus, activation of JNK by TNF*α* and the UPR may play an important role in the disruption of glucose homeostasis.

Sex-specific responses have been demonstrated in both human and rodent obesity. In humans, men have been shown to accumulate more VAT, while women accumulate more SAT, particularly in the gluteofemoral depot [33]. The visceral obesity most prevalent in males is associated with an increased risk of metabolic dysfunction and alterations in glucose homeostasis when compared to the SAT or gluteofemoral obesity [33]. Similarly, female mice fed a high fat diet exhibit an increased capacity for adipocyte enlargement, as well as decreased macrophage infiltration, lower ectopic fat deposition in the liver, and later glucose tolerance impairment than male mice of the same age [34]. Differences in sex hormones may explain these gender-specific responses. The effect of IUGR on sex-specific programming of adipose dysfunction raises an important question with significant clinical implications and warrants further investigation.

Our study is not without limitations. While we demonstrated an increase in TNF*α* components and UPR activation in association with later onset glucose intolerance, we did not assess causative relationships. We also did not assess the cellular triggers of increased TNF*α* and UPR activation. Future studies examining macrophage infiltration, ROS production, and serum free fatty acid levels will be important to elucidate the direct cause of increased of TNF*α* signaling and UPR activation. Similarly, a mechanistic understanding of the causative relationships between TNF*α* and the UPR in the subsequent development of glucose intolerance will also be important.

In conclusion, IUGR induces adipose dysfunction, inflammation, and the UPR prior to the onset of obesity in SAT of male rat pups. We speculate that these events increase the risk for insulin resistance, cardiovascular disease, and other metabolic diseases later in life.

## Figures and Tables

**Table 1:** IUGR decreased body weight (gm) in rat pups on d21 and d45 (mean ± SEM).

Postnatal age (days)	Male		Female	
	Control	IUGR	Control	IUGR
d21	62 ± 1.3	55 ± 1.9*	57 ± 1.2	51 ± 0.9*
d45	278 ± 6	261 ± 10*	195 ± 17	193 ± 14

*Different from age- and sex-matched control group, *P* < 0.05.
